# The power of many: multi-database approach to understand the relationship between statins and glaucoma

**DOI:** 10.1038/s41433-025-04003-w

**Published:** 2025-09-16

**Authors:** Mohamed S. Sayed, Mohammad Ayoubi, Mohamed M. Khodeiry, Abdelrahman M. Elhusseiny, Richard K. Lee

**Affiliations:** 1 Eye Institute, Cleveland Clinic Abu Dhabi, Abu Dhabi, UAE; 2 University of Miami Miller School of Medicine, Miami, FL USA; 3 Bascom Palmer Eye Institute, University of Miami Miller School of Medicine, Miami, FL USA; 4 Department of Ophthalmology and Visual Sciences, University of Louisville, Louisville, KY USA; 5 Department of Ophthalmology, Harvey and Bernice Jones Eye Institute, University of Arkansas for Medical Sciences, Little Rock, AR USA

**Keywords:** Epidemiology, Glaucoma

## Big data in ophthalmology

Modern medical research relies on a range of study designs to answer questions about disease risk, progression, and treatment response. Traditional epidemiologic studies excel in control and precision, while Big Data approaches leverage large-scale, multi-centre datasets to enable broader analyses across diverse populations. Beyond just size, Big Data captures real-world clinical practice patterns and population-level trends that reflect actual healthcare delivery, while enabling advanced statistical methods for complex analyses.

Glaucoma exemplifies how studies can reach conflicting conclusions, and how “Big Data” might help reconcile them. Take the association between statin use and glaucoma as a case in point. Initial longitudinal studies by Stein et al. suggested that statins confer a modest protective effect against open-angle glaucoma (OAG), with long-term use associated with reduced glaucoma risk [1]. Yet a subsequent pooled analysis of three large cohorts (over 130,000 participants) found no significant association between statin exposure and incident primary OAG (POAG) [2]. More recently, an analysis from the National Institute of Health *All of Us* dataset even reported *higher* glaucoma prevalence among statin users, particularly in certain subgroups (e.g. adults aged 60–69 with hyperlipidaemia) [3]. Faced with such disparate findings—one study suggesting a preventive benefit, another neutrality, and yet another indicating potential harm—clinicians are left with considerable uncertainty regarding the interpretation and application of this evidence.

Our group’s recent study adds a new dimension to this debate [4]. By analysing a large multi-centre electronic health record (EHR) network, we found that the statin–glaucoma relationship may depend on a patient self-reported racial and ethnic background. In our cohort of over 300,000 hyperlipidaemia patients, statin use was associated with a significantly lower risk of ocular hypertension and OAG in non-Hispanic White and Black patients, whereas in Asian and Hispanic patients the protective effect was minimal or only evident with longer-term use [4]. In other words, the impact of statins on glaucoma risk was not uniform across populations. These results suggest a unifying hypothesis: earlier studies might have disagreed because they examined different populations. For instance, Stein’s predominantly White cohort showed benefit from statins [1], whereas the null findings by Kang et al. could reflect a more mixed population or different exposure duration [2]. Meanwhile, the *All of Us*-based study (with a diverse sample) noted an apparent harm signal, which could relate to unmeasured confounders (like cholesterol levels or healthcare-seeking behaviour) or perhaps specific subpopulations where statins do not help [3]. It is important to note that Lee et al. [3] assessed glaucoma risk without differentiating between its subtypes, whereas both our study [4] and that of Stein et al. [1] specifically examined the development of open-angle glaucoma as the primary outcome.

The notion that a treatment’s effect can vary by demographics is well precedented in medicine—for example, the efficacy of certain blood pressure medications differs by race. Black patients tend to respond better to diuretics and calcium-channel blockers, whereas beta-blockers are slightly less effective on average in Blacks than in Whites [5]. Similarly, the efficacy of statins seems to vary in different populations. Genetic polymorphisms, such as those in ABCG2 and ABCA1, alter statin metabolism and plasma concentrations, particularly in East Asian individuals. Thus, if a protective effect of statins exists only in some ethnic groups (or under certain conditions), studies lacking those groups could reach different conclusions than studies enriched for them. Recognising this possibility is the first step toward resolving the conflict.

The past decade has seen the emergence of several such platforms in medicine and ophthalmology. The Veterans Affairs Million Veteran Programme (MVP), for instance, has enroled over 900,000 United States (U.S.) veterans to create one of the world’s largest biobanks [6]. The United Kingdom (UK) Biobank, another landmark resource, follows half a million adults with deep phenotyping—including comprehensive health questionnaires, physical exams, blood biomarkers, multimodal imaging, and genome-wide genotyping for every participant [7]. Likewise, the NIH *All of Us* Research Programme is building a cohort of one million Americans with an explicit emphasis on diversity (over 50% of participants are from racial or ethnic minorities), linking EHR, genomic data, surveys, and wearable device data to capture a rich array of health determinants [8].

Understanding the inherent characteristics and limitations of these datasets is also crucial. For example, the Intelligent Research in Sight (IRIS) Registry primarily captures data from community-based ophthalmology practices, while the Sight Outcomes Research Collaborative (SOURCE) draws primarily from hospital systems [9, 10]. Although initiatives like *All of Us* deliberately prioritise demographic diversity, systematic gaps continue to persist across all major ophthalmic datasets [8]. Rural populations, individuals with limited healthcare access, and certain socioeconomic groups remain chronically underrepresented. A possible solution could be federated learning networks that connect underrepresented healthcare settings, allowing their patient populations to contribute to large-scale analyses and create more comprehensive population coverage without the barriers of traditional data sharing agreements. Addressing these gaps by including underserved populations could significantly enhance the external validity and broad applicability of big data findings.

While Big Data holds immense promise in resolving controversies such as the statin–glaucoma relationship, it is not without limitations. Challenges such as misclassification, residual confounding, and overreliance on administrative codes can undermine data accuracy. Additionally, race-stratified findings must be interpreted cautiously, given the complex interplay of genetics, environment, and social determinants. To address these issues, Table 1 summarises key limitations in Big Data studies and emerging solutions, including phenotype standardisation, federated data structures, and causal inference techniques such as Mendelian randomisation and pragmatic trials.

**Table 1:** Common limitations of big data approaches in ophthalmology and potential solutions.

Challenge	Description	Emerging solutions
Misclassification of outcomes	Reliance on ICD codes may inaccurately label disease status or fail to differentiate glaucoma subtypes	Integration of structured EHR data with clinical text and imaging; use of standardised phenotyping algorithms
Residual confounding	Unmeasured factors (e.g., healthcare utilisation, adherence) may bias associations	Propensity score methods, instrumental variable analysis, Mendelian randomisation
Underrepresentation of key populations	Minoritised groups, rural patients, and those with limited access are often underrepresented	Federated learning networks, targeted data recruitment, demographic weighting
Variability in clinical definitions	Lack of standardised definitions for outcomes and exposures across datasets	Adoption of shared ontologies; efforts in ophthalmic data harmonisation
Multiple comparisons in subgroup analyses	Increased risk of type I error when analysing small strata	Bayesian hierarchical models; pre-specification and correction for multiple testing

*ICD* International Classification of Diseases, *EHR* Electronic Health Record.

Looking forward, future efforts should focus on improving population representation, harmonising clinical definitions, and integrating novel analytical frameworks that better account for bias and support causal conclusions. By complementing traditional epidemiologic approaches with well-designed Big Data studies, researchers can generate more reliable, equitable, and clinically actionable insights. As the field continues to evolve, a thoughtful application of these resources will be essential to guide evidence-based care. With accuracy, inclusivity, and innovation, Big Data can help ophthalmology move beyond uncertainty toward more precise and personalised disease prevention.
